# Noisy: Identification of problematic columns in multiple sequence alignments

**DOI:** 10.1186/1748-7188-3-7

**Published:** 2008-06-24

**Authors:** Andreas WM Dress, Christoph Flamm, Guido Fritzsch, Stefan Grünewald, Matthias Kruspe, Sonja J Prohaska, Peter F Stadler

**Affiliations:** 1 Department of Combinatorics and Geometry (DCG), MPG/CAS Partner Institute for Computational Biology (PICB), Shanghai Institutes for Biological Sciences (SIBS), Shanghai, PR China; 2 Max Planck Institute for Mathematics in the Sciences, Inselstrasse 22 -26, D 04103 Leipzig, Germany; 3 Institut für Theoretische Chemie und Molekulare Strukturbiologie Universität Wien, Währingerstraße 17, A-1090 Wien, Austria; 4 Institute of Biology II: Zoologie, Molekulare Evolution und Systematik der Tiere, University of Leipzig, Talstrasse 33, D-04103 Leipzig, Germany; 5 Interdisciplinary Center for Bioinformatics, Universität Leipzig, Härtelstraße 16-18, D-04107 Leipzig, Germany; 6 Santa Fe Institute, 1399 Hyde Park Rd., Santa Fe NM 87501, USA; 7 Biomedical Informatics, Arizona State University, PO-Box 878809, Tempe, AZ 85287, USA; 8 Bioinformatics Group, Department of Computer Science, Universität Leipzig, Härtelstraße 16-18, D-04107 Leipzig, Germany; 9 RNomics Group, Fraunhofer Institut for Cell Therapy and Immunology (IZI), Perlickstraße 1, D-04103 Leipzig, Germany

## Abstract

**Motivation:**

Sequence-based methods for phylogenetic reconstruction from (nucleic acid) sequence data are notoriously plagued by two effects: homoplasies and alignment errors. Large evolutionary distances imply a large number of homoplastic sites. As most protein-coding genes show dramatic variations in substitution rates that are not uncorrelated across the sequence, this often leads to a patchwork pattern of (i) phylogenetically informative and (ii) effectively randomized regions. In highly variable regions, furthermore, alignment errors accumulate resulting in sometimes misleading signals in phylogenetic reconstruction.

**Results:**

We present here a method that, based on assessing the distribution of character states along a cyclic ordering of the taxa, allows the identification of phylogenetically uninformative homoplastic sites in a multiple sequence alignment. Removal of these sites appears to improve the performance of phylogenetic reconstruction algorithms as measured by various indices of "tree quality". In particular, we obtain more stable trees due to the exclusion of phylogenetically incompatible sites that most likely represent strongly randomized characters.

**Software:**

The computer program noisy implements this approach. It can be employed to improving phylogenetic reconstruction capability with quite a considerable success rate whenever (1) the average bootstrap support obtained from the original alignment is low, and (2) there are sufficiently many taxa in the data set – at least, say, 12 to 15 taxa. The software can be obtained under the GNU Public License from .

## Introduction

Sequence conservation in real data often varies dramatically along multiple sequence alignments ranging from constant sites to sequence positions that have effectively been randomized. In the context of phylogenetic reconstruction, homoplastic sites – i.e., those in which the same character appears in two distinct sequences by convergence (back- and parallel-mutation) rather than by common ancestry – pose a well-known problem. Depending on the method, in the worst case they present a misleading signal (as in the case of parsimony methods), at best they increase the noise in the data (as in most distance-based methods). In addition, alignment errors producing effectively "homoplastic sites" are known from simulation studies to decrease the accuracy of the reconstruction of tree topologies [1]. For real data, ref. [2] showed that alignment errors can change the result of a phylogenetic analysis significantly.

Consequently, one may try to improve the accuracy of tree reconstruction by eliminating all putative homoplastic or otherwise corrupted sites, e.g., all third-codon positions of protein-coding genes. However, since the quality of tree reconstruction decreases with decreasing sequence length, it is important not to remove too many sites from an alignment. For example, while certain first- and second-codon positions may be essentially constant (and therefore phylogenetically useless) or hyper-variable (and hence even misleading), third-codon positions of protein-coding genes can well be informative and should not be just discarded as such [3]. There is no consensus in the literature regarding the tolerance of phylogenetic methods to multiple substitutions [4,5].

Given any alignment, it is therefore of interest to detect clearly homoplastic or otherwise corrupted sites from putative phylogenetically informative sites so that they – and no others – can be excluded or down-weighted. The complication with such an endeavor, however, is that, formally, homoplasy is defined relative to a given phylogenetic tree while it is exactly a phylogenetic tree that molecular phylogenetics is attempting to derive from an alignment. Thus, care has to be taken that homoplasy detection does not implicitly presuppose a phylogenetic tree later to be derived from the same data.

Character compatibility [6] can be used to identify fast evolving sites [7,8]. Two alignment columns are compatible if there is a phylogenetic tree for which both columns are homoplasy-free. Fast-evolving sites are expected to be incompatible with more columns than slowly evolving ones. Consequently, sites that have more incompatibilities than random sites are removed from the alignment [9]. If there are conflicting signals in the data, sites supporting the weaker one tend to be removed. Several methods simply delete the most highly variable alignment columns [10,11], the S-F approach [12] presupposes well-established groups and evaluates within-group variation relative to between-groups variation.

In this contribution, we present a new method for determining "noisy" sites in an alignment that is not *a priori *restricted to tree-like data. It is based on the observation that distances derived from pairwise sequence comparisons give rise to fairly robust **circular **split systems [13] which, in turn, are consistent with a large number of possible tree topologies [14,15]. We only use the cyclic ordering of the taxa which some methods constructing circular split systems compute in their first step, not a reconstructed tree, to assess the degree to which an alignment site is randomized. A computer program, called noisy, implements this approach.

## Trees, metrics, and weighted split systems

Let *X *denote a finite set of *n *taxa. A *split S *= *A*|$\bar{A}$ = $\bar{A}$|*A *is a bipartition of the set *X *of taxa, i.e., a partition of *X *into two disjoint, non-empty subsets *A *and $\bar{A}$. Two such splits *A*_1_|${\bar{A}}_{1}$ and *A*_2_|${\bar{A}}_{2}$ of *X *are called *compatible *if one of the four intersections *A*_1 _∩ *A*_2_, *A*_1 _∩ ${\bar{A}}_{2}$, ${\bar{A}}_{1}$ ∩ *A*_2 _and ${\bar{A}}_{1}$ ∩ ${\bar{A}}_{2}$ is empty. A split system is compatible if every pair of splits is compatible.

It is a well known result that compatible split systems on *X *are in 1-1 correspondence with the so-called *X*-trees [16], i.e., finite trees *T *= (*V, E*) with vertex set *V *and edge set *E *endowed with a map from *X *into *V *whose image contains (at least) all vertices of degree less than 3.

More specifically, this correspondence is given by associating

(i) to any edge *e *∈ *E *of such a tree *T*, the bipartition *S*_*e *_of *X *into those two subsets of *X *that are mapped into the (exactly) two distinct connected components of the graph obtained from *T *by deleting the edge *e*,

(ii) and to *T *the collection S(*T*) := {*S*_*e *_: e ∈ *E*} of all such splits.

Associating a positive weight *α*_*S *_to any such split *S *= *A*|$\bar{A}$ (e.g., the length of the edge *e *in case every edge in the tree is endowed with some predefined positive length and *S *= *S*_*e *_holds), one can define the associated metric *d *on *X *by associating, to any two taxa *x, y *in *X*, the term

(1) $$d(x,y):=\sum_{S\in S(T)}\alpha_{S}\delta_{S}(x,y)$$

where one puts, for any split *S *= *A*|$\bar{A}$ ∈ S(*T*) and all *x, y *∈ *X*, *δ*_*S *_(*x, y*) := 0 if *x, y *∈ *A *or *x, y *∈ $\bar{A}$ holds, and *δ*_*S *_(*x, y*) := 1 otherwise (i.e., if *x *and *y *are *separated *by the split *S*) implying that *d*(*x, y*) is the total length of the unique path from (the image of) *x *to (the image of) *y *relative to the given family of split weights ${\left(\alpha_{S}\right)}_{S\in S(T)}$.

It is our goal to detect homoplasy **without **first determining a tree; thus we have to admit more general split systems. We use circular split systems which we will introduce next.

## Noise detection using circular orderings

A split system S is *circular *if the points in *X *(i.e., the taxa) can be arranged on a circle so that each split *S *∈ S is induced by a division of that circle into two arcs by deleting two of its (unlabeled) points. In this case, the circular ordering is said to *represent *the split system.

It is easy to verify that compatible split systems are circular (actually, every planar drawing of an *X*-tree provides such a circular ordering), and that circular split systems are *weakly compatible *– i.e., *A*_1 _∩ *A*_2 _∩ *A*_3_, *A*_1 _∩ ${\bar{A}}_{2}$ ∩ ${\bar{A}}_{3}$, ${\bar{A}}_{1}$ ∩ *A*_2 _∩ ${\bar{A}}_{3}$ or ${\bar{A}}_{1}$ ∩ ${\bar{A}}_{2}$ ∩ *A*_3 _is empty for any three splits *A*_1_|${\bar{A}}_{1}$, *A*_2_|${\bar{A}}_{2}$, *A*_3_|${\bar{A}}_{3}$ in a circular split system, cf. [13]. Any distance constructed from a weighted circular split system is called a "circular" (or Kalmanson) metric.

It has been observed that phylogenetic distance data are often circular or at most mildly non-circular [14,17,18]. Starting from a suitable distance measure, we can construct a circular split system from an alignment without significantly prejudicing later tree constructions since the circular split system still represents essentially unfiltered data.

Prescribing a circular order *C*, of course, restricts the possible phylogenetic trees. Indeed, the fraction $\frac{2^{n-2}}{(n-1)!}$ of fully resolved trees compatible with a given ordering goes to zero with the number *n *of leaves going to infinity. On the other hand, given any circular ordering, there are quite a few - more precisely, there are exactly $\frac{1}{n-1}\left(\begin{array}{c} 2n-4\\ n-2 \end{array}\right)$  - fully resolved trees that are compatible with it [15]. Furthermore, if the true phylgenetic tree *T *is not compatible with the pre-supposed circular order *C*, we can still expect that *T *will be compatible with a circular order *C' *that differs from *C *by only a small number of breakpoints – after all, we will compute *C *from the data that have evolved according to *T *. Hence, characters that are informative for *T *(and thus for *C*^'^) can be expected not to "look random" when arranged according to *C *instead of *C*^'^. Thus, circular orders appear to offer a robust way to assess the "phylogenetic information content" of characters (alignment columns) without strongly prejudicing the subsequent tree construction. Circular split systems can be obtained in various ways. The computationally most straightforward approach is the Neighbor-Net algorithm [19] that starts from a distance matrix. It computes the circular splits using an agglomerative procedure.

An alternative approach starts from weighted quartets. To this end, one first computes a weight for each quartet, i.e., each pair of two pairs of taxa, {{*a, b*} {*c, d*}}. This quartet weight is interpreted as the support for the hypothesis that {*a, b*} and {*c, d*} are separated by an edge in the correct phylogenetic tree. Quartet weights can be obtained in various ways. In the *quartet-mapping *approach [20] for example, one starts with an alignment of four sequences and defines the weight of a given quartet to be the fraction of alignment sites (columns) in which *a *= *b *≠ *c *= *d*. One may modify this score by adding 1/2 for every additional column in which *a *= *b *≠ *c, d *or *c *= *d *≠ *a, b *holds. Quartet weights can also be derived directly from distances (although, in this case, it seems preferably to use the faster Neighbor-Net approach). A more sophisticated weighting scheme uses "expected branch lengths", i.e. the product of the posterior likelihood and the maximum likelihood branch length of the interior edge of the corresponding quartet tree.

The quartet {{*a, b*} {*c, d*}} is said to be *realized *by a cyclic ordering of *X *if the straight line connecting *a *and *b *and the straight line connecting *c *and *d *do not intersect in the interior of the circle. There is a circular split system represented by a given cyclic ordering that contains a split that separates *a *and *b *from *c *and *d *if and only if {{*a, b*} {*c, d*}} is realized by that cyclic ordering. Hence, to ensure that as much quartet information as possible is represented, QNet [21] tries to find a cyclic ordering such that the sum of the weights of all realized quartets is maximal.

Both, Neighbor-Net and QNet, use the same agglomeration process to construct a cyclic ordering. While Neighbor-Net tries to group those taxa close to each other that have a small distance, QNet tries to construct a cyclic ordering that maximizes the sum of the weights of the quartets it realizes. Hence, both methods construct cyclic orderings with the property that groups of phylogenetically closely related taxa tend to assemble along an arc. Neighbor-Net and QNet are both *consistent*, i.e., if the distances or quartet weights correspond to a circular split system, then they find a cyclic ordering that represents that split system [22,23].

For our purpose, the important property of the circular orderings computed by Neighbor-Net and Qnet is that phylogenetically more closely related taxa are preferentially placed closer together in the cyclic ordering. Thus, if a character *χ *= *χ*_*i *_(defined by some *alignment site i *in a given alignment) is phylogenetically "useful", its character states will appear "clustered" along the cyclic ordering, independent of the details of the branching order in individual subtrees. In contrast, if a character is completely randomized, we will observe that character states are randomly arranged along the cycle. The amount of clustering can be easily quantified by the number *ν *= *ν *(*C*, *χ*) of adjacent distinct character states along the cycle *C*. We have *ν *= 0 for constant sites, and *ν *≥ 2 for all non-constant sites. This number has to be compared with the numbers expected for a random distribution of character values along the cycle, given the overall distribution of the character values of *χ *. It is in principle possible to compute this distribution.

For two-state characters, a formula for the number of options to putting *v *ones and *n *- *v *zeros on a cycle of length *n *such that there are 2*k *≤ min{2*v*, 2(*n *- *v*)} breakpoints (an odd number of breakpoints is impossible) is easy to derive: There are $\frac{n}{k}\left(\begin{array}{c} v-1\\ k-1 \end{array}\right)\left(\begin{array}{c} n-v-1\\ k-1 \end{array}\right)$ such options. The explicit evaluation of such expressions is relatively expensive, however. Alternatively, very large tables would need to be pre-computed and stored to accomodate large numbers of sequences and/or character states.

Therefore, we opted for a shuffling procedure instead: we randomly generate a cyclic ordering *C' *of the same character states (and their respective frequencies) as those in *C *and compute the fraction *q *= *q*(*C, χ*) of randomized samples with *ν*(*C', χ*) > *ν*(*C, χ*). Hence we can interpret *q *as a reliability measure for the phylogenetic information contained in the alignment site (relative to *C*). Note that we obtain *q *= 0 for constant and singleton sites, which are phylogenetically uninformative and *q *≊ 0.5 for effectively randomized sites. Sites with *q *≪ 0.5 are "worse" then random and contradict the given cyclic ordering while support for the ordering is found in sites with *q *≫ 0.5.

The program noisy executes the following commands:

1. Compute the cyclic ordering *C *from the input data using either Qnet or NeighborNet.

2. For each character *χ*

• Compute the number *ν*(*C, χ*) of break points.

• Compute *N *random cyclic orderings *C'*.

• For each cyclic ordering compute *ν *(*C', χ*).

• Compute the fraction *q*(*C, χ*) of random orderings with *ν*(*C', χ*) > *ν*(*C, χ*).

3. If *q*(*C, χ*) is smaller than a given threshold, then remove the character *χ*.

The program noisy is implemented in ISO C++ and the source code is available for download from . In a first phase, a cyclic ordering of the taxa set is computed. For this purpose, noisy includes the corresponding subset of routines from the NeighborNet [19] and the QNet [21] packages. Subsequently, a reliability score *q *for each character is calculated. The number of character-state alterations is counted and compared to the observed count in random shufflings. The uniform pseudo-random number generator Mersenne Twister [24] is used to generate the random shufflings.

**Figure 1:**
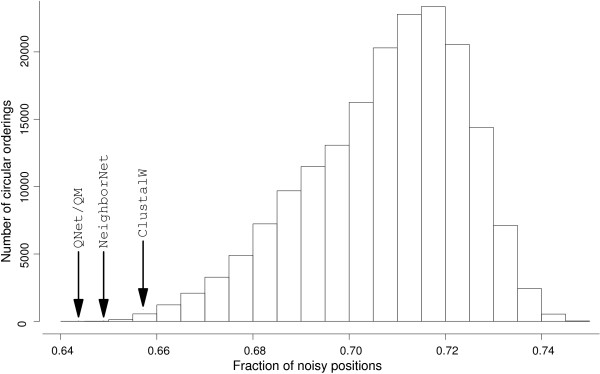
**Number of cyclic orderings of a set 10 complete mitochondrial genomes with a prescribed fraction of "noisy" characters, i.e., *q*(*C, χ*) ≤ 0.8)**. The cyclic orderings computed by NeighborNet or QNet indeed essentially minimize the fraction of putative randomized alignment sites. At least in this example, QNet with quartet-mapping-derived quartet weights performs best. "ClustalW" refers to the circular ordering implicitly constructed by ClustalW from its guide tree which determines the order in which sequences and profiles are combined to yield the final alignment.

**Figure 2:**
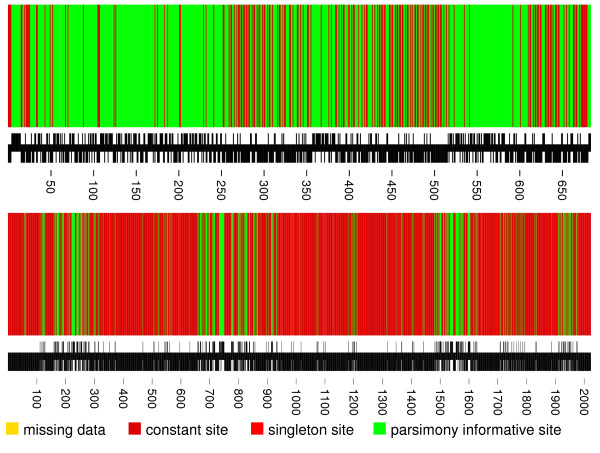
Distribution of homoplastic sites for the mitochondrial atp6 gene of squamata (2047 positions, above) and for 18S RNA of Coleoptera from an analysis of [37] (684 positions, below). In terms of quality, the two data sets are very different. While the majority of sites in atp6 are parsimony informative and approximately one third of the sites have a reliability score above *q*_cutoff _= 0.8, this is clearly not the case for the data set by [37] where most of the sites are constant or unreliable. The black bar below the alignment indicates whether the *q*-value of the corresponding position is above (upper half) or below (lower half) the cutoff value. Note that only green positions have a chance to having *q*-value above the cutoff value.

In order to assess whether the cyclic orderings obtained using QNet and NeighborNet reduce the fraction of uninterpretable variation, we performed the following randomization experiment. Given an alignment, we generated all possible cyclic orderings and computed the fraction *r *of sites with *q *> 0.8 among all variable sites in the alignment. As shown in Fig. 1, QNet and NeighborNet nearly minimize the fraction of "noisy" alignment sites for the 10 squamate mitochondria. The program noisy exports a Postscript file, visualizing the quality of the sites of the reordered input alignment (see Fig. 2), recording their reliability score as xy-data, and containing a modified alignment for further analysis in which sites with reliability *q *<*q*_*cutoff *_are removed. Fig. 2 shows typical examples for the distribution of alignment sites with low and high reliability scores *q*.

## Computational results

**Figure 3:**
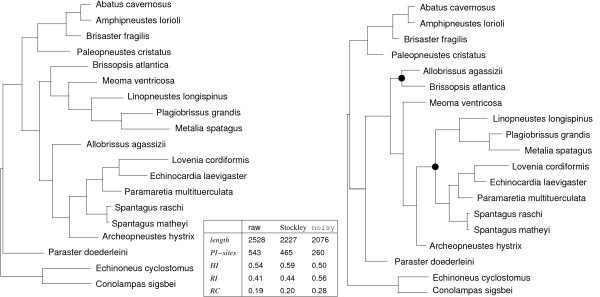
MP trees of spatangoid sea urchins from combined 28S rRNA, 16S rRNA, and mitochondrial COI sequences [25]. L.h.s. from original data, r.h.s. from a reduced alignment with cutoff *q *= 0.8. The latter tree matches the biological expectation and fits very well with those reported in [25] that were obtained from a manually reduced alignment. In particular, the noisy-reduced MP tree correctly shows *Brissopsis *and *Allobrissus *as sister groups and it correctly identifies the large monophyletic clade consisting of the *Linopneustes*/*Metalia *and *Lovenia*/*Spatangus *groups to the exclusion of *Meoma *and *Archeopneustes*. These major improvements are marked with a bullet. The included table compares the stability indices (HI = homoplasy index, RC = rescaled consistency index, RI = retention index) between the complete (unprocessed), Stockley's manually improved, and the noisy-reduced alignment.

As an example for the effect of removing "noisy" sites, we consider a data set of combined 28S rRNA, 16S rRNA, and mitochondrial COI sequences of spatangoid sea urchins that was reported to have a high level of homoplasy [25]. The "raw" sequence alignments lead to phylogenetic trees that differ significantly for different methods and disagree substantially with morphology-based results. As discussed in the original paper [25], manual removal of homoplastic sites improved the trees considerably. The application of noisy with cutoff *q*_cutoff _= 0.8, on the other hand, leads to consistent results for all methods including MP (Maximum Parsimony) that agree with the best trees reported in [25]. In Fig. 3 we present the MP trees for the unedited and the noisy-reduced alignments.

In order to assess to what extent the removal of unreliable sites from real and simulated alignments affects the commonly used measures of tree stability, we consider the *q*_cutoff_-dependency of the most common indices for tree quality. Phylogenies were computed using maximum parsimony and neighbor joining (Kimura 2-parameter model) as implemented in PAUP 4.0b10 [26]. Scaled log-likelihood score (i.e., the log likelihood divided by the length of the alignment), homoplasy index (HI) [27], rescaled consistency index (RC) [28], and average bootstrap support (over all internal vertices) were used to assess the tree stability while topological changes were described by split distance [29]. Data sets are available for download as part of the Electronic supplement [30].

**Figure 4:**
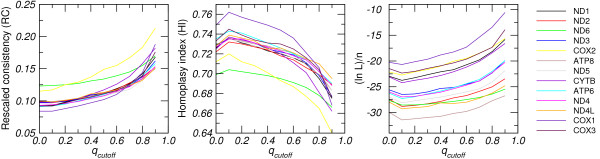
**Dependency of tree-quality indices on the cut-off value *q*_cutoff _for the protein-coding mitochondrial genes from all 31 currently available squamata**. The stability of the trees is measured by the scaled log likelihood (ln *L*)/*n*, the homoplasy index (HI) [27], and the rescaled consistency index (RC) [28] as computed by PAUP 4.0b10 [26]. Data sets are alignments (supplied in the electronic supplement) of individual mitochondrial protein-coding genes. They vary in size (from about 170 to 1800 nt) and randomization.

Fig. 4 summarizes the data for alignments of mitochondrial protein-coding genes. The other data sets show the same qualitative behavior. Table 1 shows that the fraction of effectively randomized sites varies considerably (from 26% to 37%) between different proteins even in the relatively benign case of mitochondrial genomes [31]. As expected, the homoplasy index is significantly reduced while the rescaled consistency index and the scaled log-likelihood values increases with increasing values of *q*_cutoff_. While the tree-stability indices improve consistently indicating that the reconstructions become more stable, the absolute values of the quality indices nevertheless depend strongly on the size and quality of the input alignments.

**Table 1:** Randomized sites (at *q*_cutoff _= 0.8) in the 13 different individual protein-coding genes within the 31 currently available complete mitochondrial genomes of squamata. *sngl*: number of singleton positions, *%rnd*: percentage of randomized variable sites.

Gene	length	sngl	*q *≥ 0.8	%rnd
atp6	684	42	405	34.65
atp8	171	7	108	32.75
cox1	1536	88	1008	28.65
cox2	672	34	443	29.02
cox3	786	45	516	28.63
cytb	1131	74	676	33.69
nd1	942	44	589	32.80
nd2	1032	63	626	33.24
nd3	345	11	222	32.46
nd4	1371	65	831	34.65
nd4l	288	16	183	30.90
nd5	1803	103	1040	36.61
nd6	540	25	373	26.30

Ref. [32] suggested another way to estimate the phylogenetic information content of an alignment. To this end, they determined the skewness-test statistics *g*_1 _of the corresponding tree-length distribution. We analyzed the data with the random-tree option implemented in PAUP 4.0b10 [26]. For the data matrices, we estimated 100.000 trees at random from all possible tree topologies (replacements allowed). The results are consistent with the tree statistics discussed above. As expected, we observe that *g*_1 _becomes more negative with increasing values of *q*_cutoff_, at least as long as one does not start to remove too many informative sites (data not shown).

An alternative measure for the stability of a phylogenetic reconstruction is the bootstrap support for trees – resulting, in our case, from neighbor joining [33]. In some cases, the improvement can be substantial, as in the case of a Dytiscus data set provided in the supplement, where the average bootstrap support increases from 47 to 68 (neighbor-joining trees computed using PAUP 4.0b10 and 2000 bootstrap replicates [34,35]). In benign data sets, however, the changes are typically small.

**Figure 5:**
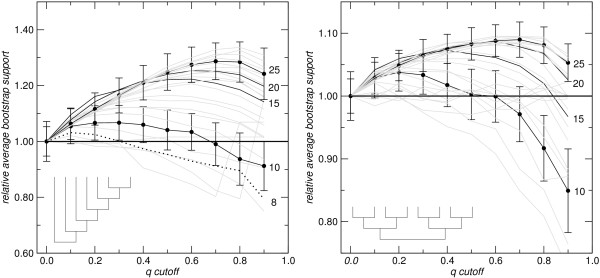
**The relative average bootstrap support of phylogenetic trees is computed as the ratio of the average bootstrap support for the modified alignments divided by the bootstrap support obtained from the original alignment**. Values larger than 1 indicate an increase in tree robustness. The curves show a distinct maximum that depends on the number of taxa and the topology of the tree. The maximum improvement increases with the number of taxa (indicated on the right margin of both panels for the highlighted curves). For clarity, error bars obtained from 100 replicates are shown only for *N *= 10 and *N *= 25 taxa. The tree topologies, caterpillar trees on the left and balanced trees on the right, are depicted by the insets.

In order to study the effect of removing putative homoplastic sites in a more systematic way, we generated artificial data sets for caterpillar and balanced trees with 4 to 29 taxa using dawg [36]. Fig. 5 shows the variation of the bootstrap support relative to the cutoff value *q*. Pairs of caterpillar and balanced trees with the same number of taxa were constructed such that (a) all leaves have the same evolutionary distance from the root and (b) all internal edges as well as all edges leading to leaves with maximal depth (maximal number of internal nodes on the path to the root) have the same "unit length". This unit length is set to 0.4 substitutions per site for the balanced trees. In the caterpillar trees the "unit length" is scaled such that the total length equals that of the balanced tree with the same number of species. For each tree, we then used dawg to generate 100 independent alignments using the following parameters: alignment length 800 nt, GTR model with *γ *= 0.5 and *ι *= 0.1, and dawg's default substitution matrix for the GTR model. We observe a pronounced maximum of bootstrap support whose position and height, however, depends strongly on both, the number of taxa and the topology of the tree. For small values of *q*_cutoff_, alignment stability increases because only the most "noisy" sites are removed. (In contrast, tree stability decreases immediately when randomly chosen alignment columns are removed; data not shown). For large values of *q*_cutoff_, tree stability starts to decrease again because noisy starts to remove too many informative sites. Empirically, we found for large data sets that *q*_cutoff _≈ 0.8 is a good compromise between these two effects. In principle, an optimal cut-off value could be estimated, provided a well-curated training set was available. For small data sets, with less than 15 taxa, we found no improvements except for rather small *q*_cutoff _values reflecting the fact that, for small data sets, there are not too many possibilities for the values of *ν*(*C, χ*) implying that noisy should be used only for at least moderately large data sets.

In general, the caterpillar trees admit larger improvements in bootstrap support than the balanced ones. We remark that the balanced trees are almost correctly reconstructed while the caterpillar trees are poorly reconstructed, in particular at the deep nodes (data not shown).

A systematic analysis of the effects of tree shape and branch length distributions will be given elsewhere. We will also discuss in that note how our algorithm can be used to deal with the alignment problems addressed in [2].

## Discussion

It has been argued repeatedly that saturated – homoplastic – characters are detrimental to phylogeny reconstruction and, thus, should be removed from multiple sequence alignments [5]. Since homoplasy is defined relative to the unknown true tree, it is not obvious, however, how to reliably identify the homoplastic characters without prior knowledge of that tree. In this note, we show that cyclic orderings that can be obtained robustly, e.g., from pairwise distance data, without detailed knowledge of the correct phylogenetic relationships can be employed for this task. Given a circular ordering that is consistent with the phylogeny, the variation of character states of a given site along the circle is used to determine the (putative) degree of its randomization. This information can then be used to prune the sequence alignment. The computer program noisy that is publicly available from the authors' website implements this procedure.

High rates of substitutions not equally distributed among sites in the sequences caused, e.g., by sequence constraints due to environmental pressure can produce a considerable amount of phylogenetic noise in the data and so-called "bad" and phylogenetically misleading alignments. Such alignments can be improved by increasing the signal-to-noise ratio through exclusion of noisy sites. Alignment modifications like concatenation of conserved blocks, known to improve phylogenetic analysis and carried out manually, are common practice. However, manual improvements are almost impossible for large-size alignments, and typically make it hard to reproduce the results later on. Furthermore, they are not immune to the effects of wishful thinking. On the other hand, a method such as noisy provides an essentially deterministic and unbiased solution.

It is important to note that "good" alignments cannot be further improved by the reduction of alignment length. While especially distance-based methods for phylogenetic reconstruction are fairly robust and can tolerate a good fraction of phylogenetically uninformative sites (see in particular [1]), a high absolute number of informative sites is necessary to obtain reliable trees.

The analysis of artificial data sets allows us to propose a set of simple rules that allow the user to decide under which conditions it makes sense to use noisy to process multiple sequence alignments prior to using them for phylogenetic reconstruction:

(1) If the original alignment already yields trees with very high average bootstrap support, there is nothing to be gained from our method.

(2) Data-sets with less than about 10 taxa are unlikely to improve.

(3) The cutoff value of *q *depends on the tree topology and in particular on the number of taxa. It pays to determine the maximum of the gain as a function of *q *and to use the corresponding optimal cutoff value.

The analysis of several published data sets shows that removal of randomized sites consistently leads to more stable trees, irrespective of the method used for phylogeny reconstruction (neighbor joining, maximum parsimony, or maximum likelihood). While in benign data sets, the effects on consistency indices, likelihood score, or bootstrap support are typically small and we do not observe changes in the reconstructed tree topologies, the effects of removing homoplastic sites can become dramatic for poor data sets, as the example of the *Cox1 *genes of Dytiscus demonstrates. More importantly, in some cases, the reconstructed tree topologies can be improved as well, see e.g. the example of the sea urchin phylogeny in Fig. 3.

Our approach removes randomized sites from a pre-computed alignment. In contrast to manual manipulation of alignments, reducing data sets using noisy is transparent and easy to reproduce. Assuming that randomized sites are, at best, phylogenetically uninformative or, in the worst case, just misleading, we propose a new way of phylogenetic reconstruction that is based on minimizing the number of randomized sites. Detecting homoplastic characters using circular orderings allows us to explore a two-stage approach: In the first step, one would construct a circular ordering that minimizes the fraction of "noisy" sites (as in Fig. 1). In the second step, one would then construct the tree implied by the alignment obtained after elimination of all sites that appear to be highly randomized relative to that circular ordering.

## Competing interests

The authors declare that they have no competing interests.

## Authors' contributions

GF and SJP initiated this study and performed the computations, SG provided a prototype of Qnet, AWMD and PFS suggested the algorithmic approach, CF and MK implemented noisy, and all authors closely collaborated on the interpretation of the results and the preparation of the manuscript.

## References

[B1] Ogden TH, Rosenberg M (2006). Multiple Sequence Alignment Accuracy and Phylogenetic Inference. Syst Biol.

[B2] Landan G, Graur D (2007). Heads or tails: a simple reliability check for multiple sequence alignments. Mol Biol Evol.

[B3] Björklund M (1999). Are Third Positions Really That Bad? A Test Using Vertebrate Cytochrome b. Cladistics.

[B4] Yang Z (1998). On the best evolutionary rate for phylogenetic analysis. Syst Biol.

[B5] Wägele JW (2005). Foundations of Phylogenetic Systematics.

[B6] Le Quesne WJ (1969). A method of selection of characters in numerical taxonomy. Syst Zool.

[B7] Wilkinson M (1992). Consensus compatibility and missing data in phylogenetic inference. PhD thesis.

[B8] Meachem CA (1994). Phylogenetic relationships at the basal radiation of angiosperms: further study by probability of character compatibility. Syst Bot.

[B9] Pisani D (2004). Identifying and removing fast-evolving sites using compatibility analysis: an example from the arthropoda. Syst Biol.

[B10] Yang Z (1994). Maximum likelihood phylogenetic estimation from DNA sequences with variable rates over sites: approximate methods. J Mol Evol.

[B11] Hansmann S, Martin W (2000). Phylogeny of 33 ribosomal and six other proteins encoded in an ancient gene cluster that is conserved across prokaryotic genomes: influence of excluding poorly alignable sites from analysis. Int J Syst Evol Microbiol.

[B12] Brinkmann H, Philippe H (1999). Archaea sister group of Bacteria? Indications from tree reconstruction artifacts in ancient phylogenies. Mol Biol Evol.

[B13] Bandelt HJ, Dress AWM (1992). A Canonical Decomposition Theory for Metrics on a Finite Set. Adv Math.

[B14] Huson DH (1998). SplitsTree: analyzing and visualizing evolutionary data. Bioinformatics.

[B15] Semple C, Steel M (2004). Cyclic permutations and evolutionary trees. Adv Appl Math.

[B16] Buneman P, Hodson FR, Kendall DG, Tautu P (1971). The Recovery of Trees from Measures of Dissimilarity. Mathematics and the Archeological and Historical Sciences.

[B17] Bandelt HJ, Dress AWM (1992). Split Decomposition: A New and Useful Approach to Phylogenetic Analysis of Distance Data. Mol Phylogenet Evol.

[B18] Wetzel R (1995). Zur Visualisierung abstrakter Ähnlichkeitsbeziehungen. PhD thesis.

[B19] Bryant D, Moulton V (2004). Neighbor-Net: An Agglomerative Method for the Construction of Phylogenetic Networks. Mol Biol Evol.

[B20] Nieselt-Struwe K, von Haeseler A (2001). Quartet-Mapping, a generalization of the likelihood mapping procedure. Mol Biol Evol.

[B21] Grünewald S, Forslund K, Dress AWM, Moulton V (2007). QNet: an agglomerative method for the construction of phylogenetic networks from weighted quartets. Mol Biol Evol.

[B22] Bryant D, Moulton V (2007). Consistency of Neighbor-Net. Alg Mol Biol.

[B23] Grünewald S, Moulton V, Spillner A (2007). Consistency of the QNet algorithm for generating planar split networks from weighted quartets. Disc Appl Math.

[B24] Matsumoto M (1998). Mersenne Twister: A 623-dimensionally equidistributed uniform pseudorandom number generator. ACM Trans Modeling Comp Simulation.

[B25] Stockley B, Smith AB, Littlewood T, Lessios HA, Mackenzie-Dodds JA (2005). Phylogenetic relationships of spatangoid sea urchins (Echinoidea): taxon sampling density and congruence between morphological and molecular estimates. Zool Scripta.

[B26] Swofford DL (2002). PAUP*: Phylogenetic Analysis Using Parsimony (* and Other Methods) Version 40b10.

[B27] Kluge AG, Farris JS (1969). Quantitative phyletics and the evolution of anurans. Syst Zool.

[B28] Farris JS (1989). The retention index and the rescaled consistency index. Cladistics.

[B29] Mailund T (2006). SplitDist – Calculating Split-Distances for Sets of Trees. Tech rep, BiRC, Univ Aarhus, Århus, DK.

[B30] Electronic Supplement. http://www.bioinf.uni-leipzig.de/Publications/SUPPLEMENTS/06-013/.

[B31] Simon C, Frati F, Beckenbach A, Crespi B, Liu H, Flook P (1994). Evolution, Weighting, and Phylogenetic Utility of Mitochondrial Gene Sequences and a Compilation of Conserved Polymerase Chain Reaction Primers. Ann Entomol Soc Am.

[B32] Hillis DM, Huelsenbeck JP (1992). Signal, Noise, and Reliability in Molecular Phylogenetic Analysis. J Hered.

[B33] Saitou N, Nei M (1987). The neighbor-joining method: a new method for reconstructing phylogenetic trees. Mol Biol Evol.

[B34] Felsenstein J (1985). Confidence limits on phylogenies: An approach using the bootstrap. Evolution.

[B35] Efron B, Halloran E, Holmes S (1996). Bootstrap confidence levels for phylogenetic trees. Proc Natl Acad Sci USA.

[B36] Cartwright R (2005). DNA Assembly With Gaps (Dawg): Simulating Sequence Evolution. Bioinformatics.

[B37] Korte A, Ribera I, Beutel RG, Bernhard D (2004). Interrelationships of Staphyliniform groups inferred from 18S and 28S rDNA sequences, with special emphasis on Hydrophiloidea (Coleoptera, Staphyliniformia). J Zool Syst Evol Research.

